# Laboratory characteristics among patients with COVID-19: a single-center experience from Khartoum, Sudan

**DOI:** 10.4314/ahs.v23i1.3

**Published:** 2023-03

**Authors:** Elmigdad Abdelgadir Ali Mohamed, Khilood Seddiq Ahmed Mohamed

**Affiliations:** 1 Alghad International Colleges for Applied Medical Sciences, Educational and Academic Affairs; 2 Sudan International University, Faculty of Medical Laboratory Sciences

**Keywords:** COVID-19, CBC, D-dimer, CRP, Sudan

## Abstract

**Background:**

COVID19 is associated with a number of laboratory characteristics and changes with different levels of prognostic significance. We report changes in lab findings between severe and non-severe COVID-19 in patients that had molecular testing of nasopharyngeal swabs in Khartoum, Sudan

**Material and Methods:**

This was a descriptive cross-sectional study, conducted from Jan to May 2021. It included 66 preidentified COVID19 patients who attended the isolation center at Jabra Hospital in Khartoum the capital city of Sudan. Participants were enrolled for CBC, D-dimer and C-Reactive Protein testing. Among these participants, 21(31.8%) had severe COVID19 pneumonia.. Data were analysed using SPSS version 24, and the independent sample t-test was used to compare severe and non-sever cases.

**Results:**

The mean values for all cases showed a mild decrease in Hb (9.53±1.83 g/dl), MCHC (28.3±2.91 g/dl); lymphocytes % (19.8 ±6.82); increased RDW-SD (50.1±5.70 fL), D-dimer (4.2±3.73 µg/ml) and CRP (107.2±61.21 mg/dl). There were significant d/span>differences in the laboratory findings between severe and non-severe COVID-19 cases in total WBCs (p value = .001), lymphocyte % (p value = .000), neutrophil % (p value=.038), RDW-SD (p value = .044), D-dimer (p value = .029) and CRP (p value = .044).

**Conclusion:**

The laboratory findings of CBC, D-dimer and CRP provide an essential contribution to predicti COVID-19 severity and prognosis.

## Introduction

Globally, as of 4:00pm CET, 28 January 2022, there had been 364,191,494 confirmed cases of COVID-19, including 5,631,457 deaths, reported to the World Health Organization, WHO. As of 27 January 2022, a total of 9,854,237,363 vaccine doses had been administered^1^. In the Sudan, from 3 January 2020 to 4:00pm CET, 28 January 2022, there had been 57,106 confirmed cases of COVID-19 with 3,422 deaths, reported to the WHO. As of 24 January 2022, a total of 3,718,299 vaccine doses have been administered^2^. By 3^rd^ March 2021, all 18 states had reported cases, with Khartoum, Aj Jazirah, and Gedaref amongst the hardest-hit. Although Khartoum State accounts for most of the reported cases in the country, the majority of COVID-19-related deaths have been reported from outside the capital. The Sudan has seen a significant increase in the number of cases being reported each day up from about 10 cases per day at the start of November 2020 to between 200-300 cases a day later in November and early December 2020. [JKT1][M2][M3]By the end of December 2020, the number of average cases per day reduced to about 200. From the second week of January 2021, the average number of daily cases went down to about 100, and from the beginning of February, it reduced further below 25 cases, according to the federal ministry of health data. Sudan's health system was under extreme stress before the pandemic and has been further stretched by added responsibility of preventing, containing and treating COVID-19. Approximately 81% of the population do not have access to a functional health center within two hours of their home and the situation is getting worse, as many clinics are closing during the pandemic. In Khartoum State alone, nearly half of the health centers closed during the pandemic, and Darfur had already closed a quarter of their facilities in 2018 due to a lack of funds and staff. The Sudan has only 184 beds in intensive care units (ICU) and approximately 160 of them have ventilators, according to WHO. Only four ICU doctors— three in Khartoum and one in Gezira State— are prepared to deal with patients infected with the virus, according to a situation report of the UN office for the coordination of humanitarian affairs^3^.

Clinically, COVID-19 is characterized by multiple manifestations, including fever, shortness of breath, persistent dry cough, chills, muscle pain, headache, loss of taste or smell, renal dysfunction, and gastrointestinal symptoms. Analogous to other similar coronaviruses. It presents a wide spectrum of signs and symptoms of varying severity; some patients are asymptomatic, and others require critical care, including ventilation, dialysis, and extracorporeal membrane oxygenation. Disease severity and mortality rates are higher in older males and individuals with other comorbidities, including obesity, diabetes, cardiovascular disease, and immunosuppression (such as cancer patients undergoing chemo- or radiotherapy and transplant patients). In contrast, women, children, and adolescents tend to be asymptomatic or mildly symptomatic, while still being contagious and contributing to viral transmission^3^–^6^.

On admission, lymphocytopenia was present in 83.2% of the patients, thrombocytopenia in 36.2%, and leukopenia in 33.7%. Most of the patients had elevated levels of C-reactive protein; less common were elevated levels of alanine aminotransferase, aspartate aminotransferase, creatine kinase, and d-dimer. Patients with severe disease had more prominent laboratory abnormalities^7^. The leading clinical signs were fever, fatigue, cough, and headache8. The prevalence of symptoms significantly varied according to age and sex. Young patients more frequently had ear, nose and throat complaints, here as elderly individuals often presented fever, fatigue and loss of appetite. Loss of smell, headache, nasal obstruction and fatigue were more prevalent in female patients 9.

Many studies noticed some impacts of viral infections on lab findings of blood cells morphology, physiology and metabolism 10. Some recent studies demonstrate multi-parameter characteristics in lab findings associated with the 2019 novel coronavirus infection. Therefore, and in light of the above, we aimed in our study to compare the changes between severe and non-severe COVID-19 in lab findings of Complete Blood Count, D-dimer, and C-Reactive Protein for COVID19 patients that pre-identified by molecular testing of nasopharyngeal swabs in our locality in the Sudan.

## Materials and Methods

### Study design

Descriptive cross-sectional study conducted in the period from Jan 2021 to May 2021.

### Sample Size

Sixty-six participants from both sexes 40(60.6%) male. Age ranged from 22 to 91 years and an average of 55±16.5 years. All participants were previously tested with RT-PCR and confirmed positive for (SARS-CoV-2).

### Excluding Criteria

Subjects with cancer, hepatic disorders, malaria-infected in the last 7 days, hematological and coagulation disorders, pregnancy, rheumatoid arthritis, and a history of drug used affect the level CBC, D-dimer, or CRP levels (aspirin, warfarin, heparin, statin, anticoagulant, and anti-platelet medications or any supplementary) were excluded from the study.

### Ethical approval

The study was approved from the ethical review committee of the Khartoum State Ministry of Health and Sudan International University. Informed consent was obtained the participants or their guardians.

## Methods

Three blood samples were collected from each participant, The first one was “EDTA” blood sample for the investigation of complete blood count “CBC” using “Sysmex XP 300” an automated 3-part differential hematology analyser uses the DC detection principle, in which the whole blood is passed between two electrodes through an aperture so narrow that only one cell can pass through at a time. The impedance changes as a cell pass through. The change in impedance is proportional to cell volume, resulting in a cell count and measure of volume^11^. The second sample was a trisodium citrated blood to extract a plasma for the D-dimer assay using the “MISPA i2” device which uses the principle of a nephelometric assay that utilizes antibody-coated latex particles. In the presence of D-dimer, the particles aggregate and light scattering increases. The increase in scattering is proportional to the amount of D-dimer in the sample^12^. While the last blood sample was used for testing the serum “CRP” using the turbidometry assay of an automated benchtop clinical chemistry analyser “Biosystem A25” in which the latex particles coated with anti-CRP are agglutinated when they react with samples that contain C-reactive protein. The latex particles agglutination is proportional to the concentration of the CRP in the sample and can be measured by turbidimetry 13.

## Statistical Analysis

Descriptive analyses were conducted with SPSS software version 24.0 and an independent sample t-test was used to compare the means between severe and non-sever cases. Differences with p values < 0.05 were considered statistically significant.

## Results

The 66 participants included 40(60.6%) males. Of the 66 cases, 21(31.8%) cases were admitted to the Intensive Care Unit “ICU” with severe 2019 novel coronavirus pneumonia and the rest 45(68.2%) were non-severe cases (Table 1).

**Table 1:** Demographic characteristic of samples

	Sever Cases	Non-sever	Cases Total
Male	13(19.7%)	27(40.9%)	40(60.6%)
Female	8(12.1%)	18(27.3%)	26(39.4%)
Total	21(31.8%)	45(68.2%)	100%

## Discussion

The comparison of CBC, D-dimer and CRP findings between severe and non-severe COVID19 cases in our study revealed significant differences in the levels of TWBCs, RDW, lymphocytes%, Neutrophils %, D-dimer and CRP with p-values (.001, .044, .000, .038, .029 and .044) respectively. Zhang et al. showed that patients with high leukocyte count (>10 × 109/L), higher neutrophil count (>7 × 109/L), and lower lymphocyte count (<0.4 × 109/L) are much more prone to severe COVID-19 pneumonia and composite endpoint “which was the admission to an intensive care unit, mechanical ventilation, or death”. Besides, higher levels of C-reactive protein (>150 mg/L) and increased D-dimer levels (>1 mg/L) are also strongly associated with an increased risk of COVID-19 pneumonia and the composite endpoint. As opposed to numerous studies, Zhang et al. showed that COVID-19 pneumonia and composite endpoint are associated with leukocytosis rather than leukopenia^3^, ^14^. Some studies reported that COVID19 patients tend to have normal or decreased white blood cell counts, lymphopenia, or thrombocytopenia^3^, ^15^, ^16^.

Our study revealed significantly increased levels of leukocytes and neutrophil % and decreased lymphocyte % in the mean values of severe cases compared to non-sever cases which were also parallel with many studies such as Soraya et.al and Pozdnyakova et.al^17^, ^18^. Laboratory data have shown that most patients had a decrease in lymphocyte count, Also, laboratory data recorded an elevation in leukocytes^19^. Soraya and Ulhaq noticed: “A twenty-six studies in the second analysis showed significantly lower lymphocyte count”^18^. While Pourbagheri-Sigaroodi et al. reviewed: “A data from 15 published articles reflecting the values of blood cell count and differential percentages of lymphocytes and neutrophils from patients with severe/non-severe COVID-19 are as presented, while lymphopenia is a prominent finding in most patients”^20^. Pozdnyakova et.al noticed that: “All patients with COVID-19 demonstrated striking numeric and morphologic WBC changes, which were different between mild and severe disease states. More severe disease was associated with significant neutrophilia and lymphopenia, which was intensified in critically ill patients. Abnormal WBC morphology, most pronounced in monocytes and lymphocytes, was associated with the milder disease; the changes were lost with disease progression. Between COVID-19-positive and COVID-19-negative ICU patients, significant differences in morphology-associated research parameters were indicative of changes due to the severe acute respiratory syndrome coronavirus 2 virus, including higher RNA content in monocytes, lower RNA content in lymphocytes, and smaller hypogranular neutrophils” 17.

The significant difference in “RDW” between severe and non-sever groups in our study was well explained in the recent study of Saeedian et.al which was reported statistically significant correlations between high levels of RDW and developing secondary infections and longer hospitalization^21^.

Elevated D-dimers further support the occurrence of coagulopathy and it is an important indicator of disease progression. It was previously established that inflammation-related parameters are highly elevated in acute phases. COVID-19 makes no exception to this rule, whereby the C-reactive protein (CRP) is increased in the sera of these patients with different values20. The levels of D-dimer and CRP in severe and critical patients were significantly higher than those in common patients and the levels of CRP in critical patients were significantly higher than those in severe patients. Pearson correlation analysis showed that the levels of D-dimer were positively correlated with the levels of CRP which is concluded that the D-dimer and CRP are highly expressed in severe and critical patients and both D-dimer and CRP have certain clinical value in evaluating the severity and prognosis of COVID-19 22.

However, a study by Thomas et.al suggested alterations in RBC membrane integrity in COVID-19 patients leading to increased susceptibility to oxidant stress-induced lysis with a minor increase in the MCV and the absence of significant changes in the RBC count, HCT, or other clinical hematological parameters^23^.

## Limitations

The current research has some limitations that need to be addressed. First, our study includes a small number of participants (n = 66). Second, we present results from a single-center, so it was not a multicenter clinical trial or nationwide survey. Third, the small number of participants does not allow to be made in-depth statistical analysis. In this regard, the results and conclusions should be interpreted with caution. Despite these limitations, this work presents results which will be useful for clinical practice.

## Conclusion

In summary, Laboratory characteristics of CBC, D-dimer and CRP among the COVID19 patients revealed significant differences in the parameters of TWBCs, Lymphocytes, Neutrophils, RDW, D-dimer and CRP which were playing an essential role in predicating COVID19 severity and provide prognostic significance assistance to support medical decision making at a time when they are urgently needed.

## Figures and Tables

**Table 2:** Descriptive results of CBC, D-dimer and CRP

	Minimum	Maximum	Mean ± (STD.DV)	Ref. Range
TWBCs X10^3^ / µl	4	14	6.39 ± (1.85)	4 - 11
RBCs X10^6^ / µl	3.2	5.23	4.22 ± (1.12)	M: 4.7 - 6.1 F: 4.2 – 5.4
Hb (g/dl)	5.4	14.3	9.53 ± (1.83) *	M: 13.5 - 17 F: 11.5 - 15.5
HCT %	24.3	50	39.6 ± (4.62)	M: 40% - 50% F: 36% - 45%
PLTs X10^3^ / µl	17	460	265 ± (118.50)	150 - 450
MCV "fl"	28.5	109.2	88.7 ± (9.11)	80 - 100
MCH "pg/cell"	15.3	36.9	29.1 ± (3.40)	25.4 - 34.6
MCHC g/dl	22.2	49.3	28.3 ± (2.91) *	33.4 - 35.5
Lymph %	6	39	19.8 ± (6.82) *	20% - 40%
Neutrophil %	37.2	88.4	53.3 ± (5.88)	40% - 75%
RDW "fL"	41.5	86	50.1 ± (5.70) *	39 - 46
MPV "fL"	7.8	12.7	10.4 ± (9.26)	7 - 12
D dimer (µg/ml)	0.34	11.02	4.2 ± (3.73) *	0 – 0.5
CRP (mg/l)	9.4	281	107.2 ± (61.21) *	8 – 10

* According to the hospital lab reference ranges the mean values of hemoglobin "Hb", mean corpuscular hemoglobin concentration "MCHC" and Lymphocytes % were mildly decreased. The means of red cell distribution width "RDW", D-dimer and C reactive protein "CRP" were increased.

**Table 3:** Comparison of the CBC, D dimer and CRP findings between severe COVID-19 and non-sever COVID-19 groups

	Sever COVID-19 Mean ± (STD.DV)	Non- Sever COVID-19 Mean ± (STD.DV)	*P.* Value (sig.)
TWBCs X10^3^ / µl	7.96 ± (1.83)	5.62 ± (1.80)	.001*
RBCs X10^6^ / µl	4.13 ± (0.57)	4.32 ± (0.58)	0.167
Hb g/dl	9.45 ± (1.38)	9.66 ± (1.11)	0.506
HCT %	39.3 ± (4.74)	39.7 ± (3.55)	0.708
PLTs X10^3^/µl	271 ± (125)	250 ± (94)	0.426
MCV "fL"	87.5 ± (10.4)	90.2 ± (5.3)	0.176
MCH "pg/cell"	28.4 ± (1.6)	29.4 ± (3.9)	0.183
MCHC "g/dl"	27.8 ± (1.2)	28.9 ± (3.2)	0.082
Lymph %	14.0 ± (6.3)	29.2 ± (7.0)	.000*
Neutrophil %	54.6 ± (7.6)	51.3 ± (2.8)	.038*
RDW "fL"	48.9 ± (4.8)	51.4 ± (6.3)	.044*
MPV "fL"	10.9 ± (9.4)	9.8 ± (0.7)	0.544
D dimer µg/ml	5.11 ± (3.2)	3.3 ± (4.3)	.029*
CRP mg/l	116.9 ± (72.5)	96.5± (40.2)	.044*

*P ≤. 0.05 is significant

* Significant differences in the parameters of TWBCs, Lymphocytes, Neutrophils, RDW, D-dimer and CRP
